# The Effect of Plant Inbreeding and Stoichiometry on Interactions with Herbivores in Nature: *Echinacea angustifolia* and Its Specialist Aphid

**DOI:** 10.1371/journal.pone.0024762

**Published:** 2011-09-13

**Authors:** Caroline E. Ridley, Helen H. Hangelbroek, Stuart Wagenius, John Stanton-Geddes, Ruth G. Shaw

**Affiliations:** 1 Department of Ecology, Evolution, and Behavior, University of Minnesota, St. Paul, Minnesota, United States of America; 2 Division of Plant Science and Conservation, Chicago Botanic Garden, Glencoe, Illinois, United States of America; University of Utah, United States of America

## Abstract

Fragmentation of once widespread communities may alter interspecific interactions by changing genetic composition of interacting populations as well as their abundances and spatial distributions. In a long-term study of a fragmented population of *Echinacea angustifolia*, a perennial plant native to the North American prairie, we investigated influences on its interaction with a specialist aphid and tending ants. We grew plant progeny of sib-matings (*I*), and of random pairings within (*W*) and between (*B*) seven remnants in a common field within 8 km of the source remnants. During the fifth growing season, we determined each plant's burden of aphids and ants, as well as its size and foliar elemental composition (C, N, P). We also assayed composition (C, N) of aphids and ants. Early in the season, progeny from genotypic classes *B* and *I* were twice as likely to harbor aphids, and in greater abundance, than genotypic class *W*; aphid loads were inversely related to foliar concentration of P and positively related to leaf N and plant size. At the end of the season, aphid loads were indistinguishable among genotypic classes. Ant abundance tracked aphid abundance throughout the season but showed no direct relationship with plant traits. Through its potential to alter the genotypic composition of remnant populations of *Echinacea*, fragmentation can increase *Echinacea*'s susceptibility to herbivory by its specialist aphid and, in turn, perturb the abundance and distribution of aphids.

## Introduction

Understanding the evolution of interactions between species is of growing importance in modern landscapes. Habitat destruction differentially affects abundances of interacting species, and fragmentation of populations alters breeding patterns and, thereby, genetic composition. The immediate genetic effects of destruction and fragmentation of habitat include reduction in the effective size and genetic diversity of populations due to increased mating between related neighbors. In plant populations, fragmentation may also increase mating between distant isolates, either through increase in frequency of long-distance pollinator flights [1] or through human-mediated movement of plants, whether inadvertent or via intentional restoration efforts [2]. Thus, beyond the direct effect of landscape use on the numerical composition of communities of interacting species, changes in genotypic composition may affect interactions, and, consequently, the dynamics of communities and the ongoing evolution of their component populations.

Conspecific plants may differ in their susceptibility to and effects on herbivores via multiple mechanisms [3]. First, plant apparency to herbivores may vary, through traits such as size, phenology, and production of volatile compounds. Second, once detected by herbivores, plants may deter them or limit their growth through defensive traits such as trichomes and secondary compounds [4]. Third, nutritional quality of hosts, of which elemental composition is a major component, may affect growth of consumer populations [5] and their evolution, as demonstrated in studies of *Daphnia* in chemostats [6], [7]. Moreover, other interacting species (*e.g.*, mutualistic ants) may be directly influenced by plant traits, such as volatile compounds, or indirectly through traits of the herbivores.

Traits that influence species' interactions are typically genetically variable. Consequently, different crossing patterns in natural populations, from matings between self or sib parents (inbred) to matings between distantly related parents, are likely to affect these traits and the inter-species interactions that they mediate. For example, plant inbreeding can modulate the effects of herbivores [8], [9], [10], [11]. The effect of inbreeding on fitness is generally deleterious [12], whereas the effects of crossing between populations may be neutral, may increase fitness (heterosis), or may decrease fitness (outbreeding depression), depending on the generation evaluated, the distance between populations, and other considerations [13]. For some plant populations, fitness is maximized at intermediate crossing distances [14], [15], but whether intermediate outcrossing reduces plants' susceptibility to herbivory is unknown.

The abundance and diversity of herbivores are influenced by both “bottom-up” interactions with plant hosts [16], [17], [18] and by interactions with natural enemies [19], [20] and mutualists [21]. While enemies and mutualists strongly influence herbivore population size in some cases [22], effects from the bottom-up often predominate in natural settings [23], [24]. In a striking example, Johnson [25] inferred that variation among *Oenothera biennis* clones explained 29% of the variation in aphid population growth compared to less than 2% explained by predators or mutualistic ants.

We here present a study to assess the extent to which genetic consequences of fragmentation affect the interaction between the prairie perennial *Echinacea angustifolia* and its associated specialist aphid, *Aphis echinaceae*, as well as ants that tend the aphids. This study was motivated by observations that progeny resulting from random mating within remnant populations (genotypic class *W*) suffered significantly less severe leaf damage by chewing herbivores than either inbred progeny (class *I*) or those from crosses between plants from different remnants (class *B*) (Hangelbroek, Wagenius and Shaw, unpublished). Here, we evaluate differences among the three plant genotypic classes with respect to their interaction with aphids and aphid-tending ants. To investigate mechanisms mediating the interaction, we also examine the roles of plant size and elemental composition (C, N, and P) in the interaction.

## Results

### Insect occurrence

**Figure 1:**
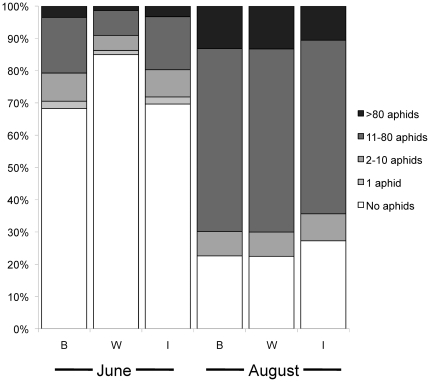
Aphid abundance observed on three plant genotypic classes. Abundance was observed in June in five abundance categories on progeny from crosses between remnants “B” (N = 96), within remnants “W” (N = 61) and between sibs “I” (N = 68).

Aphid abundance differed significantly among the genotypic classes (POLR: Likelihood ratio = 7.10, Test df = 2, P = 0.02) in June. Plants in genotypic classes *B* and *I* were twice as likely to carry aphids as *W* plants; moreover, of plants with aphids, *B* and *I* plants tended to have greater aphid loads (Fig. 1). At this early time in the season, aphids were observed on 45% of the plants overall. By August, aphid abundance had considerably increased. Aphids were observed on 72% of all plants, and the loads on individual plants were greater, no longer differing among the genotypic classes (POLR: Likelihood ratio = 0.42, Test df = 2, P = 0.81).

In June, plant size had a positive relationship with aphid abundance (POLR: Likelihood ratio = 3.40, Test df = 1, P = 0.065). In addition, leaf P had a significant negative relationship with June aphid abundance (POLR: Likelihood ratio = 13.15, Test df = 1, P<0.0002). Leaf N also significantly accounted for aphid loads, but only when leaf P was taken into account (POLR, Likelihood ratio = 9.87, Test df = 1, P<0.002); this relationship was positive. Leaf C had no significant effect on aphid loads. In a model that did not account for leaf stoichiometry, the relationship between aphid abundance and genotypic class was qualitatively similar to that found for the full model (POLR: Likelihood ratio: 6.19, Test df = 2, P = 0.045). Aphid loads varied spatially to a considerable degree (joint test of both spatial factors, POLR: Likelihood ratio = 14.61, Test df = 4, P<0.005).

In August, the relationship between aphid loads and leaf P and leaf N remained significant and in the same direction as early in the season (respectively, negative [P<0.0002] and positive [P<0.003]). The tendency of larger plants to bear greater aphid loads also persisted (P<0.084). However, by this time, there was no evidence of spatial variation in aphid loads (joint test of row and position, P>0.8).

**Figure 2:**
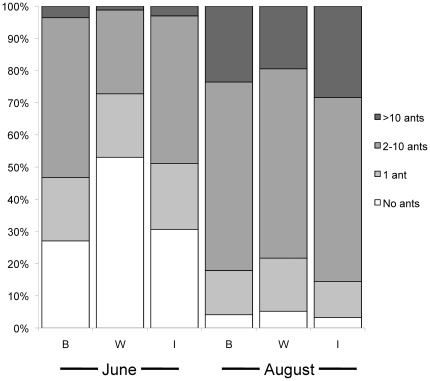
Ant abundance observed on three plant genotypic classes. Abundance was observed in four abundance categories in June on progeny from crosses between remnants “B” (N = 42), within remnants “W” (N = 21), and between sibs “I” (N = 30). Ant abundance is shown only for individuals that had at least one aphid present in June.

As with the abundance of aphids, ant loads in June tended to be lowest on plants of class *W* (Fig. 2), but the direct effect of genotypic class on ant abundance was not statistically significant (POLR: Likelihood ratio: 3.93, Test df = 2, P = 0.13). Ant abundance was, however, highly significantly related to aphid abundance (POLR: Likelihood ratio: 24.1, Test df = 3, P<0.001). Ants were observed on 36% of plants overall, and of plants that had at least one aphid, 81% also had ants. On plants where ants were present, the most commonly observed abundance was 2–10 ants.

### Elemental composition

**Figure 3:**
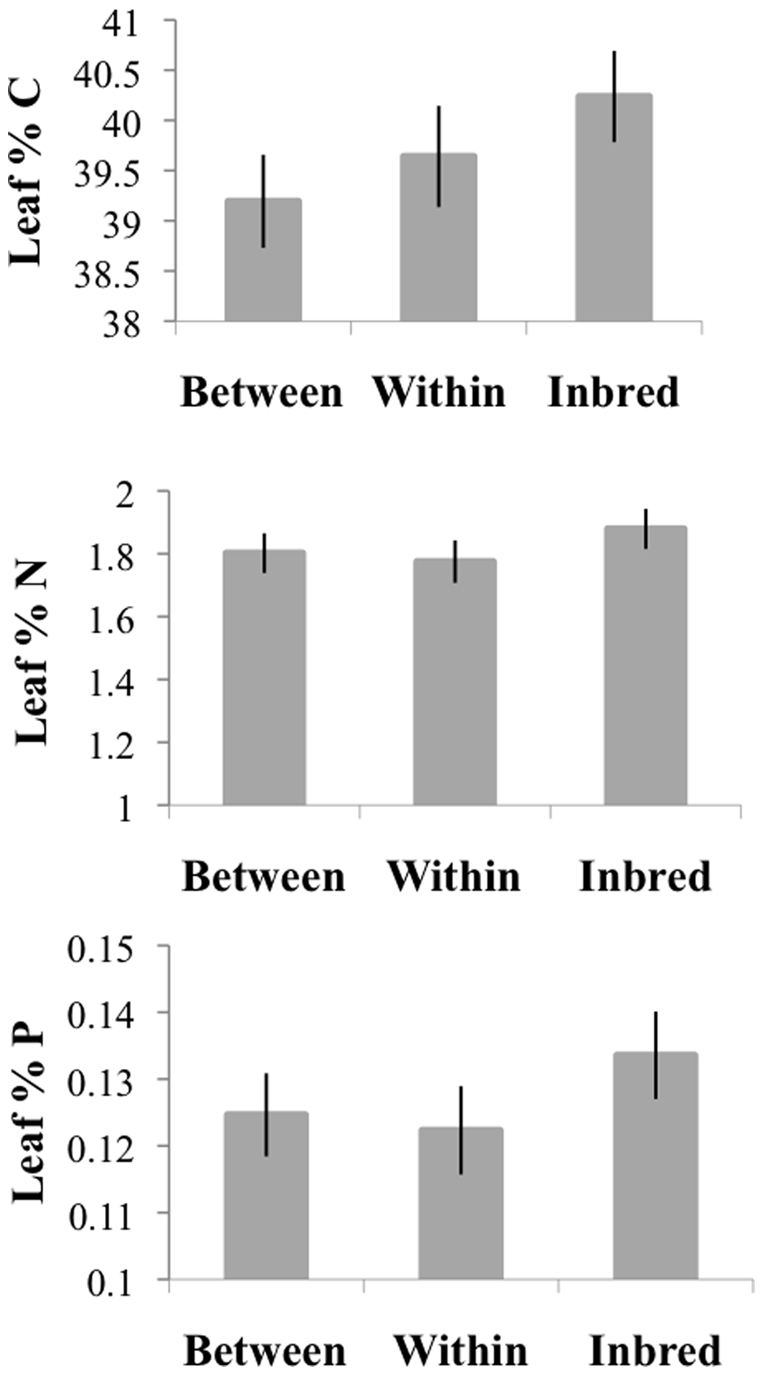
Least square means of %C, %N, and %P in leaves for each genotypic class (+/−1SE). Means are based on the univariate models presented in Table 1 and Table S1. Genotypic classes are made up of progeny from crosses between remnants “B”, within remnants “W”, and between sibs “I”. Light bars are June values and dark bars are August values. In the case of variables natural log (ln) transformed for analysis, we show back transformed values of predictions on the ln scale obtained from the models. Ln values +/−1SE were back transformed to give asymmetrical upper and lower error bounds.

Inbred plants (*I*) tended to have higher concentrations of C, N, and P in leaves in June (Table 1), but not in August (Table S1, Fig. 3). The difference among genotypic classes in June approached significance (MANOVA: Pillai-Bartlett = 0.06, Approx F_6,354_ = 1.86, P = 0.088), primarily reflecting the influence of genotypic class on leaf C and, to a lesser extent, leaf P (Table 1). Differences among genotypic classes in leaf N in June were not detectable. Elemental composition of plants varied spatially and also between the two years in which crosses were done (“crossyear”), but there was no significant effect of plant size on elemental composition (Table 1), even when genotypic class was excluded from the model (results not shown).

**Table 1:** Analysis of leaf elemental composition.

		Response							
		Leaf %C		Ln (leaf %N)		Ln (leaf %P)		Leaf CNP	
Factor	Df	Mean square	F ratio	Mean square	F ratio	Mean square	F ratio	Pillai-Bartlett	Approx F ratio
Row	3	3.35	0.734	0.029	1.032	0.067	1.167	0.043	0.868
Position	1	14.34	3.145†	0.100	3.62†	2.821	49.11**	0.273	21.97**
Crossyear	1	22.94	5.03*	0.006	0.219	0.067	1.161	0.053	3.31*
Leaf number	1	0.01	0.003	0.001	0.019	0.025	0.429	0.004	0.208
Genotypic class	2	12.47	2.735†	0.054	1.966	0.144	2.50†	0.061	1.85†
Residuals	178	4.56		0.028		0.057			

†P<0.10;

*P<0.05;

**P<0.001.

ANOVA of leaf elements (%C, %N, %P) individually, and MANOVA of combined variables (rightmost column) for leaves of *Echinacea angustifolia* in June. Similar results for August can be found in Table S1.

Plant elemental composition in June was significantly associated with the elemental composition of the aphids collected that month (Table 2). Specifically, there was a significant positive effect of leaf C and N on aphid C, and a significant positive effect of leaf P on aphid N (Table 2). The effect of genotypic class on aphid C and N concentrations approached significance (P = 0.092, Table 2). This effect largely reflects the influence of plant genotypic class on aphid C; class *W* plants harbored aphids with slightly higher C. There was no evidence of an effect of genotypic class on aphid N (Table 2). When leaf elemental composition was excluded from the model, the effect of plant genotypic class on aphid C and N was not evident (MANOVA: Pillai Bartlett = 0.075, Approx F_4, 182_ = 1.77, P = 0.14). For ants, elemental composition did not differ significantly among plant genotypic classes (MANOVA: Pillai-Bartlett = 0.048, Approx F_4,178_ = 1.09, P = 0.36) or any other factor in the analysis, including elemental composition of aphids.

**Table 2:** Analysis of aphid elemental composition.

		Response					
		Aphid %C		Aphid %N		Aphid CN	
Factor	Df	Mean square	F ratio	Mean square	F ratio	Pillai-Bartlett	Approx F ratio
Row	3	6.38	2.20†	1.68	3.22*	0.130	2.05†
Position	1	0.008	0.003	0.693	1.33	0.016	0.721
Crossyear	1	6.87	2.37	0.098	0.189	0.026	1.17
Genotypic class	2	7.07	2.44†	0.433	0.831	0.088	2.03†
Leaf number	1	4.87	1.68	0.076	0.146	0.025	1.10
Leaf %C	1	27.35	9.44**	0.000	0.000	0.103	4.98**
Ln (leaf %N)	1	22.09	7.63**	1.65	3.16†	0.092	4.39*
Ln (leaf %P)	1	3.23	0.294	3.10	5.96*	0.092	4.42*
Residuals	88	2.897		0.521			

†P<0.10;

*P<0.05;

**P<0.01.

ANOVA of aphid elements (%C, %N) individually, and MANOVA of combined variables (rightmost column) for *Aphis echinaceae*, sampled on *Echinacea angustifolia* in June.

The raw mean C∶N ratio of *Echinacea* in June for all genotypic classes was 22.1±3.8 (1 SD), while the C∶P ratio was 324.±85.9. In August, leaf C∶N was 26.1±4.7 and C∶P was 420.7±131.9. These ratios are substantially lower than typically found for terrestrial plants (∼36 and ∼970 for C∶N and C∶P, respectively, [26]), making *Echinacea* relatively rich in both N and P. The C∶N ratio of *Aphis echinaceae* was 8.6±1.1, about a third higher than the average insect herbivore at ∼6.5 [26]. The C∶N ratio for all tending ants was 4.85±0.5 and for the most common species only it was 4.92±0.5. All elemental percentages are in Table S2.

## Discussion

We have shown that plants differing in the degree of parental relatedness (genotypic classes *B*, *W*, and *I*) vary in their susceptibility to early-season infestation by a specialist aphid. In June, plant genotypic class *W*, progeny of within-remnant crosses, harbored significantly fewer aphids, compared to inbreds (*I*) and progeny of crosses between remnants (*B*). These remnant populations have been shown to be genetically differentiated with respect to fitness [27] and mean rates of inter-compatibility of plants [28]. We emphasize that the use of formal genetic crosses, rather than clonal replication of plant genotypes, eliminates nongenetic causes as explanations for differences we detected among cross types. Moreover, we underscore that the genetic differences we report are based on representative sampling of genotypic classes, each comprising progeny of multiple parents from each of seven remnant populations growing in a natural field environment.

These results parallel the observations that originally prompted this study; leaf damage from a suite of chewing insects occurred more frequently on plants from *B* and *I* crosses, compared to *W* plants, and tended to be more severe in those groups, though this difference was not consistent over years (Hangelbroek, Wagenius and Shaw, unpublished). In this study, we found differences in aphid abundance among genotypic classes early in the season. By August, aphids were more evenly distributed among the genotypic classes. Thus, as aphids became more prevalent through the season, their avoidance of *W* plants weakened, and aphid reproduction on individual plants may also have equalized abundances of aphids on the different genotypic classes. Nevertheless, preference of aphids for the inbred and outcrossed plants may differentially reduce the fitness of these plant genotypic classes.

We found that concentration of phosphorus in plants was inversely related to abundance of aphids on them, whereas there was a positive relationship with nitrogen. An intriguing finding is that these relationships between aphid loads and plant elemental composition were strong and persisted throughout the season, even as the relationship between aphid loads and genotypic class waned. In a study of a weevil on mesquite [18], an inverse relationship between weevil abundance and the ratio of foliar C to P was found at two sampling times, interestingly opposite to the relationship with P in our study, with the strength of the relationship differing between two sampling times. To our knowledge, our study is the first demonstration in an experimental context under natural conditions that intraspecific variation in elemental composition generated by crossing patterns can influence the abundance of an associated species. Aphid loads also tended to increase with plant size, as has been shown elsewhere [29].

Our findings that plant genotypic classes differ in aphid loads, even after accounting for differences in plant elemental composition and size, indicate that the genotypic classes differ in still other attributes that influence either their attractiveness to aphids or aphid population growth. For example, trichomes can impede aphid feeding [30] or protect aphids from predators [25]. The effect on herbivory of *Echinacea*'s moderately to densely hairy stems and leaves has not been examined. Physiological or biochemical attributes of plants that are only weakly associated with elemental composition may also be associated with abundance of aphids. Differences among genotypic classes in the concentration of secondary compounds, including echinacoside and various caffeic acid derivatives and alkamides, may cause them to differ in their nutritional value to and defense against aphids. In addition, the amino acid composition of an artificial lab diet has been shown to affect aphid growth and reproduction [31]. Little is known about how amino acid levels vary in nature, how they might vary among genotypic classes, or how the endosymbiotic bacteria within many aphids [32] may compensate for that variation. Finally, plant traits may affect other community members, such that natural enemies of aphids are more abundant, active, or effective on plants of the *W* genotypic class.

Aphid loads were elevated to a similar degree on *I* and *B* plants compared to *W*, but the mechanisms accounting for higher aphid loads may well differ between the two genotypic classes. We have documented severe inbreeding depression with respect to size and fitness in these plants [33], [34]. Inbreeding depression with respect to resistance to aphids may play an important role in the ∼60% loss of fitness for these progeny of sib-mating relative to progeny from random mating within remnants. Others have documented greater susceptibility of inbreds or greater fitness of herbivores on them, relative to outbreds, in studies in greenhouse and lab, but results are mixed. Delphia et al. [35] found that tobacco hornworm (*Manduca sexta*) consumed more of leafdisks from horse-nettle (*Solanum carolinense*) inbreds and also grew faster on their excised leaves than on progeny of outcrossing. Xylem-feeding spittlebug nymphs emerged as larger adults when they fed on *Mimulus guttatus* progeny of selfed matings, compared to those from outcrossing; in a second set of crosses, nymphs took longer to emerge on inbred plants [8]. Leimu et al. [11] found that snails consumed more leaf tissue of *Lychnis ( = Silene) flos-cuculi* plants from intra-population crossing than from selfing; however, the snails grew larger on inbreds.

In crosses between remnants (class *B*), susceptibility to aphid attack may be elevated if heterotic effects increase the plants' attractiveness to aphids or promotes growth of aphid populations. In this experimental population growing in nature, genotypic class *B* plants have exhibited higher survival than class *W* plants. Yet the expected value of size of *B* and *W* plants after 5 years matched closely [33], [34], as did the expected value of fitness over eight years, including the years 2006–2008, following this study, when annual reproduction had become substantial [33], [34]. An intriguing possibility is that elevated herbivory on plants of class *B*, compared to class *W*, has compromised an intrinsic advantage in growth and reproduction of genotypic class *B* over *W*. Conversely, to the extent that differential susceptibility to aphid infestation could have contributed to mortality up to the time of the study, our assessment of variation in susceptibility in the survivors may be conservative.

The strong, negative relationship between the abundance of aphids and leaf P, independent of genotypic class, may result either because aphids tend to avoid plants with high P or because their population growth is lower on those plants. Boersma and Elser [36] have shown that the growth rate of some consumers is maximized at an intermediate concentration of P (ranging from 0.25% to 1.25% depending on the species) and clearly declines at higher levels. They have argued that deleterious effects of excess P are especially likely for organisms, such as phloem feeders, that typically feed on low P substrates. In our study, P content averaged 0.3%. The negative relationship between aphid load and P content is also consistent with work showing that the cotton aphid (*Aphis gossypii*) can significantly reduce P of chrysanthemum (*Dendranthema grandiflora*) [37], a “top-down” effect [38].

Among the genotypic classes, differences in elemental concentration were modest, with inbred plants having elevated leaf C (2%) and P (∼8%), relative to the two other classes, in June. This effect did not persist through the season and was small in comparison with other studies. For instance, inbred lines of maize have 30% greater N on average than the F1 crosses between them [39]. The slightly elevated concentrations of C and P in inbreds may be due to their slower growth early in the season (inbred plants were smaller, though not significantly so). However, there was no independent effect of plant size on elemental composition. Genotypic differences dissipated as plants grew and were exposed to herbivores. Preferences of aphids for inbred plants, or greater population increase of aphids on inbreds, could equalize elemental composition if aphids differentially extract C and P.

The role of leaf elemental composition in aphid elemental composition is not straightforward, both because phloem content (which the aphids consume directly) may differ from leaves in elemental composition and because the behavior and physiology of herbivores can change in response to the quality of their food source such that dietary requirements are met and elemental homeostasis is maintained [40], [41], but see [42]. Nevertheless, we found strong associations between the concentration of C in aphids and C and N of plants and between the N in aphids and P of plants. Relative to other terrestrial plants [26], the leaves of *Echinacea* are relatively rich in N and P (Table S2), while relative to other reported insect herbivores [26], *Aphis echinaceae* is low in N.

We found that ants varied in abundance in relation to aphid abundance, but there was no significant direct effect of plant genotypic class on either abundance or elemental composition of the ants that tended the aphids. Even excluding plants that carried no aphids, within-remnant crosses most often harbored no ants. It may be that plants from class *W* tend to support too few aphids for ant attendance. We also did not find any indication that aphid stoichiometry affected the stoichiometry of the ants tending them. Using direct manipulations of diet in the lab, Kay et al. [43] showed that ant diet can affect the stoichiometry of ant workers. In the field, however, ants foraging widely likely draw honeydew from aphid colonies on multiple plants. Moreover, elemental composition of ants may more closely reflect that of aphid honeydew, rather than whole aphids.

As fragmentation increases the prevalence of mating between relatives in remnant *Echinacea* populations [28], [44], it substantially compromises plant fitness [34]. Concurrently, longer pollinator flights [1] and human-mediated movement of plants for restoration projects [2] may promote mating between plants at greater distances, producing plants genotypically similar to our class *B*. Our study shows that progeny of both kinds of matings are more susceptible to a prevalent herbivore, *Aphis echinaceae*. As extremes of crossing distances continue to increase in prairie remnants, the resulting changes in genetic composition can be expected increasingly to alter *Echinacea*'s interactions with its herbivore community and, in turn, selection on both *Echinacea* and its herbivores.

## Materials and Methods

### Ethics Statement

Permission to collect seeds from remnant populations was obtained from The Nature Conservancy and from private land owners. The Chicago Botanic Garden has no commercial interest in this research.

### Study system

*Echinacea angustifolia* (narrow-leaved purple coneflower, hereafter *Echinacea*, Asteraceae) is an herbaceous, perennial plant native to the tallgrass prairie and Great Plains of North America. It was once abundant within its extensive range, but, beginning with the arrival of European settlers about 1870 and following massive conversion of prairie to agriculture, it is now restricted to remnants of prairie. *Echinacea* individuals do not spread vegetatively; new plants emerge from seed and established plants resprout from a taproot into a rosette of basal leaves. Plants are iteroparous, with earliest flowering typically after age four and flowering in intermittent years thereafter. Recruitment of seedlings varies depending on vegetation and burning, but rarely exceeds 5% [45]. *Echinacea* is self-incompatible (SI); evidence from experimental self-pollinations suggests that SI is strictly maintained [46].

In our study area, we have observed many herbivorous insects on *Echinacea*, but an aphid apparently specializing on it is the most common. Other species observed on foliage include beetles (five families), Hemiptera (nine families including three Homopterans- *Philaenus spumarius*, *Campylenchia latipes*, and *Publilia modesta*), a dipteran (*Hylemya* sp.), and *Melanoplus* spp. grasshoppers; vouchers representing all taxa have been deposited in the Entomology Collection of the Bell Museum of Natural History. The aphid has been named as a new species, *Aphis echinaceae* Lagos [47]. We have observed these aphids, including winged migrants, on *Echinacea* leaves in the spring, as well as on stems and capitula of flowering plants, reaching densities exceeding 100 individuals on many plants in late summer. The aphids are tended by several species of ant, including *Formica obscuripes* and *Lasius spp.* Ants frequently build structures of thatch and soil on the undersides of leaves that harbor aphids.

### Experimental crosses

To investigate the role of genotypic class of plants in interactions with the aphids and ants they host, we observed and measured plants growing in a long-term experiment under natural conditions. Details on the experimental design can be found elsewhere [34]. Briefly, in fall 1995, seeds were collected from seven remnant populations of *Echinacea* in a 6400 ha study area in western Minnesota centered near 45°49′N, 95°42.5′W. In spring 1996, seeds were germinated in a growth chamber and seedlings (N = 625) were planted in a randomized array into an old field within the larger study area. The field has been overseeded with native perennial grasses, but non-native cool-season grasses and legumes dominate. The site is managed with biennial spring burns, and walkways are mowed annually. In 1999 and 2000, as plants flowered, we manually performed crosses of three types: between random individuals from different remnants (“between”), between random individuals from the same remnant population, but not sharing a maternal parent (“within”), and between maternal sibs, (*i.e.*, pairs of plants from the same maternal parent in a remnant, “inbred”). We refer to the resulting progeny as genotypic classes “*B*,” “*W*,” and “*I*” respectively. Seeds from these crosses were collected, and, in 2001, we germinated them and planted 508 seedlings in randomized locations within four rows 1 m apart with 50 cm between plants within rows in the same old field near their parents. We have demonstrated genetic differences among these remnant populations with respect to intrapopulation mating compatibility [28] and fitness expressed in common conditions [31].

### Field measurements

On June 27–29 and August 8, 2005, we inspected each leaf on each surviving plant (N = 230; 100 *B*, 61 *W*, and 69 *I*) and noted the presence and abundance of *A. echinaceae* and honeydew-collecting ants. Aphid abundance was assessed in five categories (0, 1, 2–10, 11–80, and >80 individuals), and ant abundance in four categories (0, 1, 2–10, and >10 individuals).

We collected tissue from a basal leaf in late June and early August and, in June only, collected aphids and ants from plants where they were present. Ants of several species were collected and their identity was recorded; one species accounted for 87% of the samples. In August, all preserved and dried leaf samples were ground to a fine powder with a micropestle. Ground leaf samples and whole, dried aphids and ants were sent to the Ecosystems Analysis Lab at the University of Nebraska Lincoln. They were evaluated for percent carbon and percent nitrogen using dry combustion on a Costech Analytical Elemental Combustion System 4010 (Costech Analytical Technologies Inc., Valencia, CA). Leaf samples were also evaluated for percent phosphorus colorimetrically on a Bran+Luebbe AutoAnalyzer 3 (SEAL Analytical Inc., Mequon, WI) after acid hydrolysis. Complete records for June elemental analysis were obtained for 189 plants (N = 79 *B*, 56 *W*, and 54 *I*), 110 aphid samples (N = 49 *B*, 32 *W*, and 29 *I*), and 127 ant samples (N = 55 *B*, 34 *W*, and 38 *I*). Complete records for August were obtained for 160 plants (N = 76 *B*, 42 *W*, 42 *I*).

We assessed plant size on August 4–5, as the number of leaves in the basal rosette(s).

### Analysis

To examine relationships between aphid and ant abundance and plant attributes both in June and in August, we used proportional odds logistic regression (POLR) in the MASS package of R [48], which accommodates ordered categorical responses. Aphid abundances in June and in August were modeled separately, initially with eight predictors. These included genotypic class, plant size (*i.e.*, leaf number), leaf C, leaf N, and leaf P, as the predictors of primary interest, as well as variables to account for spatial variation in the experimental field (x, y coordinates, with the row x treated as categorical, and the north-south position y treated as continuous) and the year the seed was produced (hereafter “crossyear”). We used values of leaf N and P transformed to natural logs (ln) for the June analysis, and, for the August analysis, we transformed leaf C and leaf P to natural logs, as these transformations improved the fit of the residuals from the MANOVAs (see below) to a normal distribution. “Crossyear” showed no significant effect on insect occurrence (P∼0.7) and was eliminated from further consideration. Ant abundances in June and in August were modeled with four predictors each: genotypic class, row, position, and crossyear. We complemented these analyses by examining models that added aphid abundance as a predictor. In these analyses, we included only plants where aphids were present, to prevent the absence of both aphids and ants from dominating the relationship; however, including these cases did not qualitatively change the results. We used likelihood ratio tests of the effect of plant genotypic class on aphid and ant abundances by comparing the full model with ones omitting genotypic class.

We obtained maximum likelihood estimates of expected aphid loads in each genotypic class in June and August using the full model from the proportional odds logistic regression. These estimates accounted for covariates by reporting values for a typical hypothetical individual in a middle row, with the modal leaf count, and having a median value for position, leaf C, leaf N, and leaf P [34]. We used the same approach for predicted ant loads on typical *B*, *W*, and *I* plants, using the same values for position, row.

In order to evaluate differences among plant genotypic classes in their elemental composition in June and in August, multivariate analyses of variance (MANOVA) in R [48] were conducted separately on the data early and late in the season. The response variables were percent C, N, and P in leaves (transformed as above). The main predictor of interest was genotypic class, and we also included row, position, crossyear, and size. June records were complete for 187 plants and August for 158 plants (two plants missing a size measurement in each month were excluded). Because elemental composition was not available for all plants both early and late in the season, we did not jointly analyze these measures at the two timepoints. We evaluated the effect of the predictors on the multivariate responses using the Pillai-Bartlett statistic.

## Supporting Information

Table S1 **ANOVA of leaf elements (%C, %N, %P) individually, and MANOVA of combined variables for leaves of** ***Echinacea angustifolia*** **in August.**
(DOC)

Table S2 **Mean elemental percentages (± SD) of plants, aphids and ants collected in June and August.** Means include only individuals with complete elemental information within the given month.
(DOC)
