# Supplementary material for: The Effect of Plant Inbreeding and Stoichiometry on Interactions with Herbivores in Nature: Echinacea angustifolia and Its Specialist Aphid
Source: PLoS One. 2011 Sep 13;6(9):e24762. doi: 10.1371/journal.pone.0024762 (PMC3172291; doi:10.1371/journal.pone.0024762)
Supplement: Table S1 — ANOVA of leaf elements (%C, %N, %P) individually, and MANOVA of combined variables for leaves of Echinacea angustifolia in August. (DOC) [file pone.0024762.s001.doc]

**Table S1. ANOVA of leaf elements (%C, %N, %P) individually, and MANOVA of combined variables for leaves of *Echinacea angustifolia*** in August.

|  | | **Response** | | | | | | | |
| --- | --- | --- | --- | --- | --- | --- | --- | --- | --- |
|  |  | **Ln(leaf %C)** | | **Leaf %N** | | **Ln (leaf %P)** | | **Leaf CNP** | |
| **Factor** | **Df** | **Mean square** | **F ratio** | **Mean square** | **F ratio** | **Mean square** | **F ratio** | **Pillai-Bartlett** | **Approx F ratio** |
| Row | 3 | 0.009 | 5.36* | 0.129 | 2.06 | 0.191 | 3.37* | 0.16 | 2.80* |
| Position | 1 | 0.000 | 0.007 | 0.036 | 0.577 | 5.66 | 99.78** | 0.466 | 42.75** |
| Crossyear | 1 | 0.000 | 0.057 | 0.228 | 3.65† | 0.407 | 7.17* | 0.061 | 3.16* |
| Leaf number | 1 | 0.001 | 0.413 | 0.003 | 0.044 | 0.033 | 0.585 | 0.01 | 0.485 |
| Genotypic class | 2 | 0.002 | 1.33 | 0.025 | 0.398 | 0.011 | 0.196 | 0.03 | 0.747 |
| Residuals | 149 | 0.002 |  | 0.063 |  | 0.057 |  |  |  |

† P <0.10; * P<0.05; **P<0.001
