# Supplementary material for: The Effect of Plant Inbreeding and Stoichiometry on Interactions with Herbivores in Nature: Echinacea angustifolia and Its Specialist Aphid
Source: PLoS One. 2011 Sep 13;6(9):e24762. doi: 10.1371/journal.pone.0024762 (PMC3172291; doi:10.1371/journal.pone.0024762)
Supplement: Table S2 — Mean elemental percentages (± SD) of plants, aphids and ants collected in June and August. Means include only individuals with complete elemental information within the given month. (DOC) [file pone.0024762.s002.doc]

**Table S2. Mean elemental percentages (± SD) of plants, aphids and ants collected in June and August. Means include only individuals with complete elemental information within the given month.**

| **Element** | **Leaf (June)**  **(N = 189)** | **Leaf (August)**  **(N = 160)** | **Aphid (June)**  **(N = 110)** | **Ant (June)**  **(N = 127)** |
| --- | --- | --- | --- | --- |
| C | 39.4 (2.1) | 39.1 (1.7) | 53.6 (2.0) | 48.1 (1.7) |
| N | 1.83 (0.3) | 1.55 (0.3) | 6.30 (0.8) | 10.0 (0.9) |
| P | 0.13 (0.04) | 0.10 (0.03) | NA | NA |
